# A disease similarity matrix based on the uniqueness of shared genes

**DOI:** 10.1186/s12920-017-0265-2

**Published:** 2017-05-24

**Authors:** Matthew B. Carson, Cong Liu, Yao Lu, Caiyan Jia, Hui Lu

**Affiliations:** 1 Department of Preventive Medicine, Feinberg School of Medicine, Northwestern University, 680 N Lake Shore Dr, Suite 1400, Chicago, IL 60611 USA; 2 Department of Bioengineering, University of Illinois at Chicago, 851 S Morgan St, Chicago, IL 60607 USA; 3 Center for Biomedical Informatics, Shanghai Children’s Hospital, 24 W Beijing Rd, Suite 1400, Shanghai, 200000 China; 4 Department of Computer Science, Beijing Jiaotong University, No.3 Shangyuancun, Haidian District, Beijing, 100044 China; 5 SJTU-Yale Joint Center for Biostatistics, Department of Bioinformatics and Biostatistics, Shanghai Jiaotong University, 800 Dongchuan Road, Shanghai, 200000 China

**Keywords:** Disease-disease similarity, Disease-related genes, Clustering

## Abstract

**Background:**

Complex diseases involve many genes, and these genes are often associated with several different illnesses. Disease similarity measurement can be based on shared genotype or phenotype. Quantifying relationships between genes can reveal previously unknown connections and form a reference base for therapy development and drug repurposing.

**Methods:**

Here we introduce a method to measure disease similarity that incorporates the uniqueness of shared genes. For each disease pair, we calculated the uniqueness score and constructed disease similarity matrices using OMIM and Disease Ontology annotation.

**Results:**

Using the Disease Ontology-based matrix, we identified several interesting connections between cancer and other disease and conditions such as malaria, along with studies to support our findings. We also found several high scoring pairwise relationships for which there was little or no literature support, highlighting potentially interesting connections warranting additional study.

**Conclusions:**

We developed a co-occurrence matrix based on gene uniqueness to examine the relationships between diseases from OMIM and DORIF data. Our similarity matrix can be used to identify potential disease relationships and to motivate further studies investigating the causal mechanisms in diseases.

## Background

Over the last two decades computational methods have contributed increasingly to the analysis of many diseases [1, 2]. Areas of interest include the identification and annotation of disease genes [3–5], effects of single nucleotide polymorphisms (SNPs) [6], studies on gene-drug interactions [7], semantics and ontological work [8, 9], protein interaction networks [10], and many others. Of particular interest is the investigation of the relationship between diseases in terms of genotypic and phenotypic similarity. Recent work with disease networks has revealed the interconnected nature of various diseases [11, 12], which begs the question; can we gain new knowledge of a disease such as cancer by studying “connected”, non-cancer diseases? Many diseases including obesity [13, 14], infection [15], diabetes [16], and possibly even psychological stress [17] have reported some relationship to cancer. Often the relationship type is unknown or partially known, indicating the need for further exploration of the interconnectedness of diseases. The key to understanding disease-disease similarity is to enrich the relationships with a quantifiable value and to infer new disease associations based on this enriched value.

Several strategies to measure disease similarity have been developed in previous studies. Mathur and Dinakarpandian used semantic similarity between ontological terms associated with diseases [18]. Using formal concept analysis (FCA, closely related to bi-clustering or co-clustering), Keller and colleagues identified clusters from the previous known gene-disease associations [19]. By investigating formal concepts they revealed hidden relationships between diseases based on common associated genes as well as genes associated with a common set of diseases. Suthram et al. integrated high-throughput mRNA expression data and protein-protein interaction networks to discover human disease relationships in a systematic and quantitative way. They revealed similarities between diseases by identifying functional modules among the protein-protein interactions and scoring their association with diseases.

An alternative way to define disease similarity is to not only to consider the number of the genes they shared, but also to take into account the uniqueness of shared genes or molecular features. In this study, we introduce a method for measuring similarity between pairs of diseases based on the number of genes they share only with each other. We assume that if a gene or set of genes is related to only one pair of diseases, the similarity between those two diseases should be higher than that of a pair of disease sharing gene associations with many other diseases.

## Methods

To analyze disease relationships, we built a disease co-occurrence matrix based on shared genes between each pair of diseases. We first calculated the *uniqueness* of each gene *i* as follows:

$$ {u}_i = 1-\sqrt{\frac{d_i}{d_n}} $$

where *d*_*i*_ is the number of diseases associated with each gene *i* and *d*_*n*_ is the number of diseases in the data set. Note that the fewer number of diseases related to a gene, the higher the possible uniqueness score for that gene.

Next, we created an *N* × *N* matrix. For each pair of diseases we added the uniqueness score of each shared gene:

$$ {d}_{ij}={u}_{s_1} + {u}_{s_2}+\dots +{u}_{s_n} $$

where *d*_*ij*_ is a disease pair and ${u}_{s_n}$ is the uniqueness value for each gene shared between the two. The diagonal elements of the disease co-occurrence matrix, where *i = j* for *d*_*ij*_, contain the sum of the uniqueness values for all genes related to disease *d*_*i*_.

Next, we applied *symmetric approximate minimum degree permutation* to reorder the disease co-occurrence matrix. This algorithm was developed by Stefan I. Larimore and Timothy A. Davis and incorporated into MATLAB [20]. This reordering algorithm first creates a permutation vector *p* from a symmetric positive definite matrix *A*. This permutation vector, which contains a list of reordered columns from *A*, is then used to create a new matrix *S* such that *S = (p, p)* has a sparser Cholesky factor than the original matrix *A*. The end result is that the reordered matrix *S* is less sparse near the lower diagonal and sparser near the upper diagonal. For our disease co-occurrence matrix, this effectively clusters highly related diseases in the lower right quadrant around the diagonal.

## Results

**Fig. 1:**
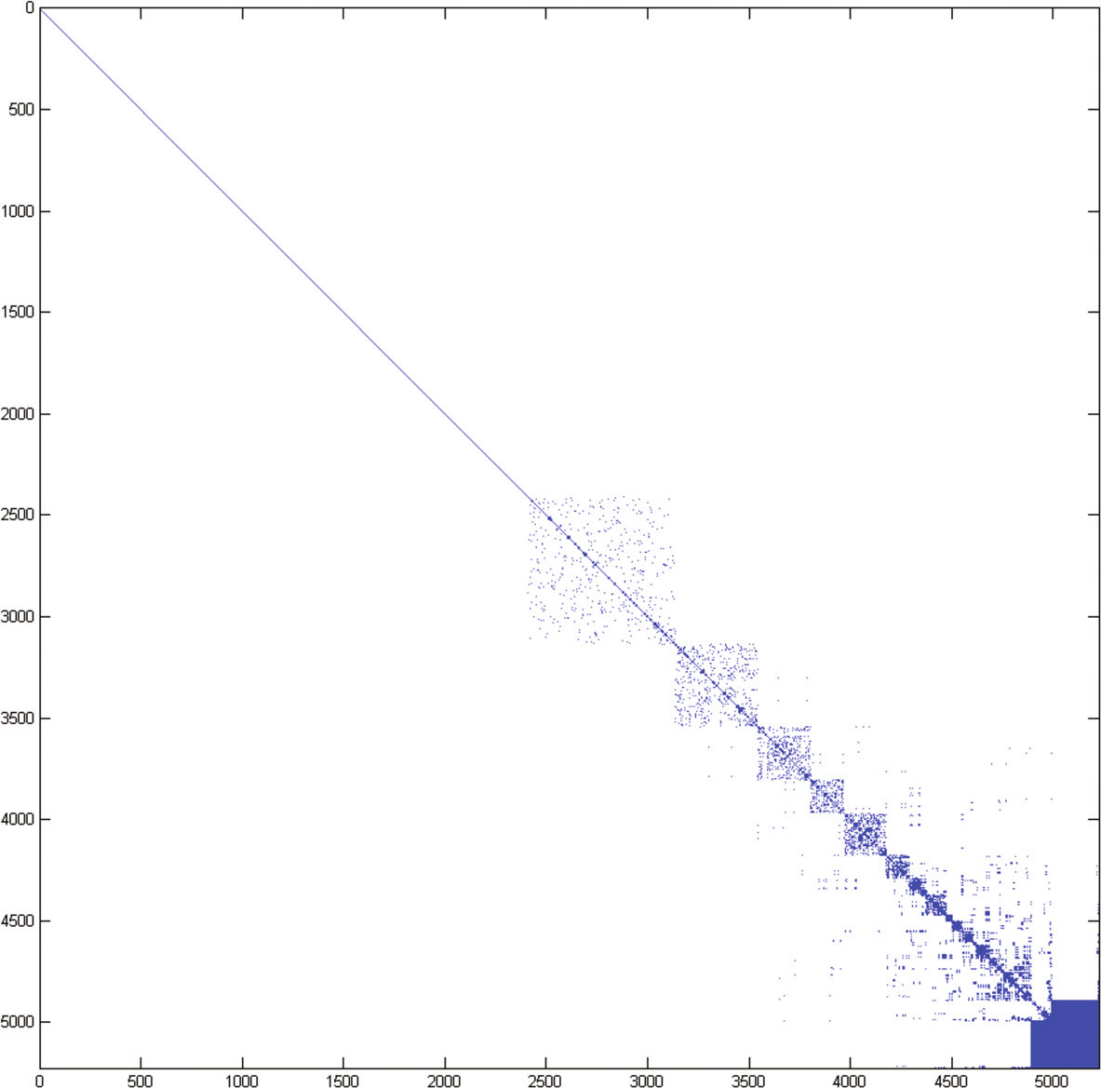
A co-occurrence matrix showing the relationship between 5,224 diseases from the OMIM MorbidMap. Matrix elements colored blue indicate a relationship between two diseases, white elements indicate no relationship. Each blue matrix element (*i, j*) contains the sum of the uniqueness values for all genes related to both *disease*_*i*_ and *disease*_*j*_ (i.e. *d*_*ij*_), while white elements are equal to 0. Diagonal elements indicate the identity relationship for each disease, i.e., the sum of the uniqueness values for all genes associated with *disease*_*i*_. This figure was created using MATLAB [20]. The disease-gene relationships were extracted from OMIM MorbidMap

We first applied this strategy to the OMIM MorbidMap database [21]. Fig. 1 shows the resulting reordered disease co-occurrence matrix for 5,224 diseases. While there are well-defined clusters, many of the cluster members are variations of the same disease phenotype or very closely related phenotypes. This is due to the high level of specificity of the OMIM disease categories. For example, the disease “46XY complete gonadal dysgenesis” is listed as two separate disease phenotypes, each with a different MIM identifier. While this distinction is important (the two phenotypes refer to mutations on different chromosomes), it does not serve our purposes in this case. We would like to see more relationships between phenotypically different diseases, and we would like very closely related phenotypes to be grouped together.

**Fig. 2:**
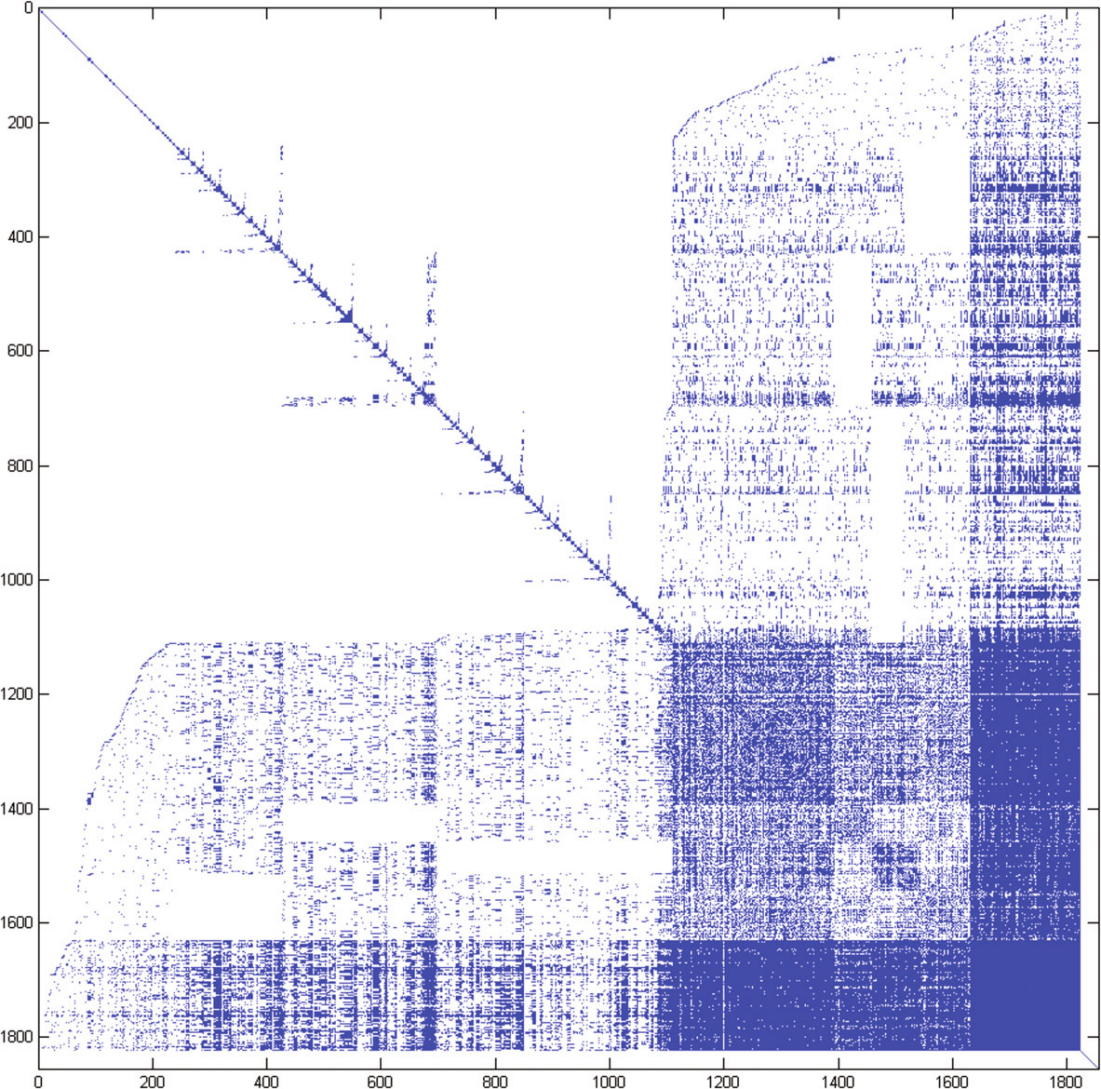
A co-occurrence matrix showing the relationship between 1,854 diseases using DORIF data. Matrix elements colored blue indicate a relationship between two diseases, white elements indicate no relationship. Each blue matrix element (*i, j*) contains the sum of the uniqueness values for genes related to both *disease*_*i*_ and *disease*_*j*_ (i.e. *d*_*ij*_), while white elements are equal to 0. Diagonal elements indicate the identity relationship for each disease, i.e., the sum of the uniqueness values for all genes associated with *disease*_*i*_. This figure was created using MATLAB [20]. The disease-gene relationships were extracted from DORIF data

To address this, we created another matrix using gene-disease relationships gathered from Disease Ontology [22] and the GeneRIFs (Gene Reference Into Function) database (http://www.ncbi.nlm.nih.gov/gene/about-generif). The Disease Ontology provides a hierarchical structure in which more specific diseases can be grouped into broader categories, which allowed us to more easily compare phenotypically divergent diseases. These data sources were used by Osborne et al. to annotate the human genome [9] for disease (referred to hereafter as DORIF, http://projects.bioinformatics.northwestern.edu/do_rif/). The data set included 5,376 genes, 1,854 diseases, and 48,436 PubMed references relating genes to diseases. The DORIF co-occurrence matrix (Fig. 2) shows the comparisons between these diseases. There are two notable differences from the OMIM matrix. First, there are noticeably more disease relationships. This is because OMIM is a curated database of Medelian diseases, while DORIF is a ‘Wiki-type’ of resource (modifiable by NCBI users willing to provide their email address), leading to a higher depth and coverage of DORIF. In addition, given the denser disease-gene network in DORIF, we would expect to observe more disease relationships. Second, the DORIF matrix appears noisier; the relationships are not as tightly clustered as they are in the OMIM matrix. OMIM is a manually curated database and thus presumably has a higher accuracy rate. A slightly noisier matrix for DORIF in comparison to OMIM is not surprising. However, if the goal is to find hidden relationships between diseases, these “gray areas” are points of interest. A closer look at the individual clusters provides some interesting information.

## Discussion

**Fig. 3:**
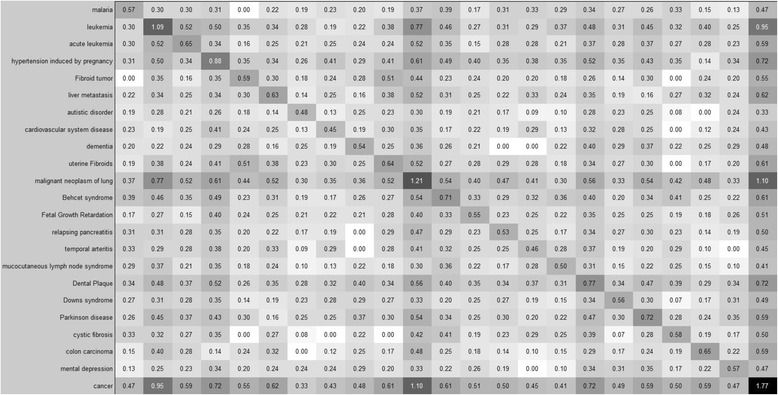
A subset of the disease co-occurrence matrix and the relationships between 23 diseases beginning with malaria (top) and ending with cancer (bottom). Disease labels for the rows apply to the columns as well. The value of each element (*i, j*)is the sum of the uniqueness values of all genes related to both *disease*_*i*_ and *disease*_*j*_ (i.e. *d*_*ij*_). Darker squares indicate a higher uniqueness value. This figure was created using MATLAB [20]. The disease-gene relationships were extracted from DORIF data

**Fig. 4:**
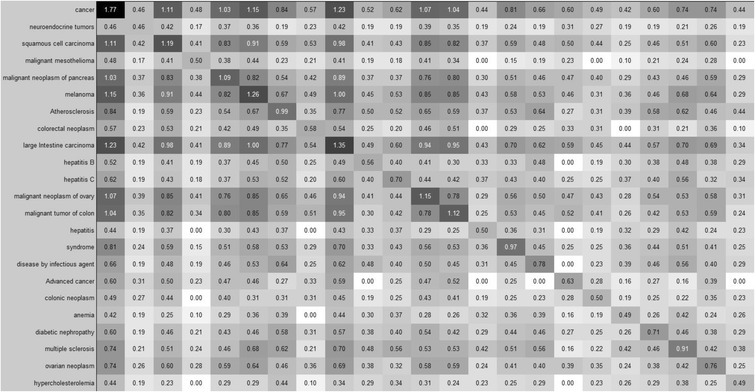
A subset of the disease co-occurrence matrix and the relationships between 23 diseases beginning with cancer (top) and ending with hypercholesterolemia (bottom). Disease labels for the rows apply to the columns as well. The value of each element (*i, j*) is the sum of the uniqueness values of all genes related to both *disease*_*i*_ and *disease*_*j*_ (i.e. *d*_*ij*_). Darker squares indicate a higher uniqueness value. This figure was created using MATLAB [20]. The disease-gene relationships were extracted from DORIF data

Figures 3 and 4 show a closer view of two different subsections of a dense cluster. Each of these figures is a 23 X 23 square submatrix of disease relationships from the solid blue cluster in the lower right-hand corner of the matrix in Fig. 2. The majority of the diseases in these submatrices are various types of cancers. There are some notable and interesting exceptions, however. For example, in the case of the relationship between malaria and cancer, the uniqueness value is close to that of those genes only related to malaria (0.47 and 0.57, respectively). Recent research provides some interesting findings about these two diseases. A clinical study showed that the mortality rate in patients with any type of cancer was increased after malarial infection [23]. Additionally, the malaria drug chloroquine has been shown to reduce tumor size in pancreatic cancer patients [24]. Another example is the relationship between hypertension induced by pregnancy and cancer. Recent work has shown that VEGF (Vascular endothelial growth factor) may be the connection. When taking anti-VEGF cancer drugs, patients develop very similar symptoms to pregnancy-induced hypertension. When VEGF expression levels are reduced in solid tumors, growth slows due to the lack of vascular development within. As a side effect, hypertensive symptoms occur [25]. Dental plaque and cancer appear to be highly related according to their uniqueness values as well; 0.77 (dental plaque) and 0.72 (dental plaque and cancer). Several past studies have made the connection between oral health and chronic illness. Recently, however, a clinical study spanning the last 24 years was released [26]. During this period, researchers followed 1,400 adults. They found that these subjects with high levels of dental plaque were 79% more likely to die prematurely from cancer. This work shows only an association between the two diseases, and thus the true nature of the relationship is yet to be discovered. Other relationships from our matrix share a high uniqueness score, but there is little or no experimental evidence linking them. For example, migraine headaches and large intestine carcinoma have a shared uniqueness score of 0.403, while migraine alone is 0.498 (not shown in figures). Despite this, we could not find research references linking the two. However, this matrix could be used to identify potential disease relationships and to motivate further study into the elucidation of causal mechanisms in disease.

## Conclusions

We developed a co-occurrence matrix based on gene uniqueness to examine the relationships between diseases from the OMIM and DORIF databases. We found examples of known disease relationships as well as connections with no available evidence. This matrix serves as a preliminary reference for identifying disease-disease associations, providing a map of the connections between diseases, and directing focus toward those associations which may not otherwise be obvious. It could also be used as a first step in drug repositioning research, directing focus to new potential protein or DNA targets. It is important to note that the purpose of this study is to provide a disease similarity matrix from the uniqueness of shared genes as a reference and that it is not meant to serve as the basis for clinical decisions in patient care.

Complex diseases such as cancer are both unique and related to other diseases, and analyzing all pairwise relationships between diseases provides new perspectives. For instance, drugs used for the treatment of related non-cancer diseases may help to treat the side effects of cancer drugs. Another example lies in the complex relationship between bacteria and cancer: bacteria can be both beneficial and cancer causing. Research on disease relationships can stimulate the development of new ideas about cancer and its relationship to infection. Additionally, this research could help clarify the mechanisms and tissue-specificity of non-cancer diseases and how they may prime the cellular environment for metastasis. We expect that in the near future, due to the availability of an enormous amount of genotypic and phenotypic data related to disease, there will be a novel view point for cancer research emerging from these studies.
